# Quantitative Bias in Illumina TruSeq and a Novel Post Amplification Barcoding Strategy for Multiplexed DNA and Small RNA Deep Sequencing

**DOI:** 10.1371/journal.pone.0026969

**Published:** 2011-10-28

**Authors:** Filip Van Nieuwerburgh, Sandra Soetaert, Katie Podshivalova, Eileen Ay-Lin Wang, Lana Schaffer, Dieter Deforce, Daniel R. Salomon, Steven R. Head, Phillip Ordoukhanian

**Affiliations:** 1 Laboratory of Pharmaceutical Biotechnology, Ghent University, Ghent, Belgium; 2 Department of Molecular and Experimental Medicine, The Scripps Research Institute, La Jolla, California, United States of America; 3 Next Generation Sequencing Core, The Scripps Research Institute, La Jolla, California, United States of America; Newcastle University, United Kingdom

## Abstract

Here we demonstrate a method for unbiased multiplexed deep sequencing of RNA and DNA libraries using a novel, efficient and adaptable barcoding strategy called Post Amplification Ligation-Mediated (PALM). PALM barcoding is performed as the very last step of library preparation, eliminating a potential barcode-induced bias and allowing the flexibility to synthesize as many barcodes as needed. We sequenced PALM barcoded micro RNA (miRNA) and DNA reference samples and evaluated the quantitative barcode-induced bias in comparison to the same reference samples prepared using the Illumina TruSeq barcoding strategy. The Illumina TruSeq small RNA strategy introduces the barcode during the PCR step using differentially barcoded primers, while the TruSeq DNA strategy introduces the barcode before the PCR step by ligation of differentially barcoded adaptors. Results show virtually no bias between the differentially barcoded miRNA and DNA samples, both for the PALM and the TruSeq sample preparation methods. We also multiplexed miRNA reference samples using a pre-PCR barcode ligation. This barcoding strategy results in significant bias.

## Introduction

Taking advantage of the increasing throughput achieved by second generation sequencing technologies, multiplexing several samples in one analysis can increase experimental throughput while reducing time and cost.

Several strategies have been described for barcoding sequencing libraries [1]–[5]. Vigneault *et al.* [4] published a miRNA barcoding protocol using ligation of 3' pre-adenylated barcoded adapter oligonucleotides as the first step of sequencing library preparation. Buermans *et al.* [5] published a miRNA sequencing protocol, introducing a barcode during PCR. Illumina recently released the TruSeq kits for multiplexed high-throughput sequencing. The Illumina TruSeq small RNA protocol introduces the barcode during the PCR step using differentially barcoded primers, while the TruSeq DNA (or messenger RNA converted to double stranded DNA) protocol introduces the barcode before the PCR step by ligation of differentially barcoded double stranded adaptors. All published methods place the barcodes within the adapters, downstream or within the PCR primer binding site or introduce the barcode during PCR. However, it is well established that multi-template PCR amplification can result in a sequence-dependent amplification bias, as some DNA species are amplified more efficiently than others [6]–[9]. For this reason, introducing barcodes near a priming site might result in a barcode-specific quantitative bias. To our knowledge, no previous publication has provided in-depth data measuring PCR amplification bias resulting from the use of barcodes.

Our initial attempts to adapt previous barcoding strategies to multiplexed sequencing of small RNA used index sequences placed at the distal end of the 5' adapter in the Illumina small RNA library protocol. Despite a number of iterations of the design we consistently failed to avoid PCR amplification bias when identical samples with different barcodes were compared. Therefore, we designed a new strategy in which we ligate both the 3' and 5' adapters, perform the RT-PCR step and then ligate the barcode after the library PCR amplification, as the last step of the library preparation. We have called this strategy Post Amplification Ligation Mediated (PALM) barcoding. In the present study, we compared the de-multiplexed quantitative results of 12 differentially PALM barcoded miRNA samples, 12 TruSeq barcoded miRNA samples and 4 miRNA samples barcoded using our above-mentioned pre-PCR barcoding strategy from the Human Brain Reference RNA (Ambion). Each pool was sequenced in a single lane on an Illumina GAIIx.

Parallel to PALM barcoding for small RNA, we also developed a PALM barcoding protocol for DNA samples or messenger RNA (mRNA) converted to double stranded DNA (dsDNA). The main difference compared to the PALM barcoding for small RNA, is the fact that double stranded adapters instead of single stranded adaptors need to be ligated before PCR. In the present study, we compared the de-multiplexed quantitative results of 12 differentially PALM barcoded DNA samples and 12 TruSeq barcoded DNA samples. Reference DNA was generated by converting *Saccharomyces cerevisiae* mRNA into double stranded DNA. Each pool was sequenced in a single lane on an Illumina HiSeq 2000.

## Methods

### PALM small RNA barcoding

**Figure 1:**
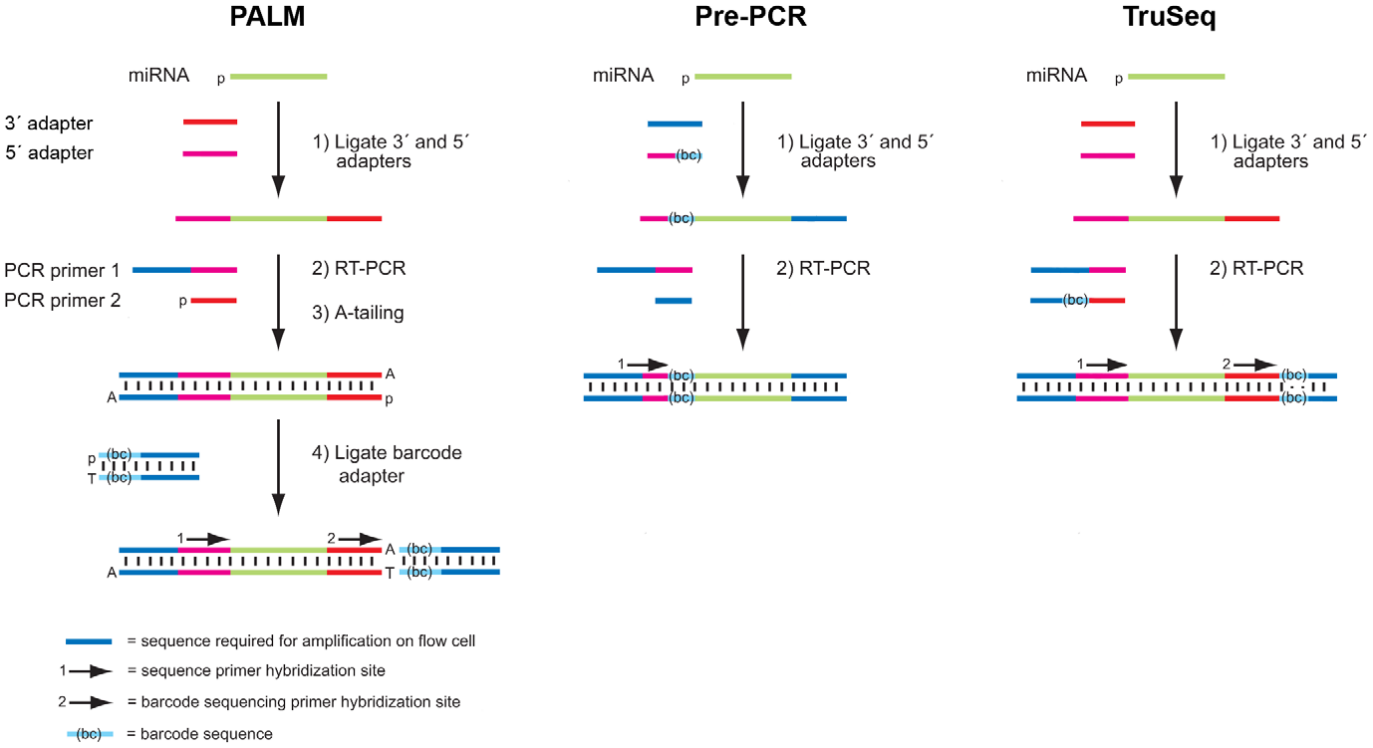
Comparative schematic of small RNA barcoding methods. The three methods start with ligation of a 3' and 5' RNA adapter to generate a substrate for RT-PCR. In the pre-PCR barcoding method, the barcode is incorporated in the 5' adapter. In the TruSeq method, the barcode is incorporated in one of the RT-PCR primers. In the PALM barcoding method, the amplified RT-PCR product is A-tailed and ligated to a T-tailed barcoded adapter.

The PALM miRNA barcoding protocol is similar to the Illumina Small RNA v1.5 Sample Preparation Guide. This protocol was modified to achieve a higher yield after the PCR amplification step using higher reaction volumes for the RT-PCR step. No extra cycles were added to the PCR reaction. The adapters used in the protocol were modified to allow for PALM barcoding and Illumina index sequencing with the Illumina multiplexing index read sequencing primer. The complete protocol, including the adapter sequences, is available in Text S1. Figure S1 shows a typical Invitrogen 4% E-gel of a Human Brain Reference RNA (Ambion) library after PCR amplification and before barcode ligation. Figure 1 shows the necessary oligonucleotide components for PALM and how they are consecutively added to the miRNA sample. The key difference with respect to the current Illumina small RNA library protocol is the addition of the barcode to the library by ligation after PCR amplification. After ligation of the barcode, no further purification of the library is required. The library is quantified using analysis of area under the peaks with a BioAnalyzer 2100 (Agilent) to determine the correct loading concentration for subsequent sequencing.

### Pre-PCR barcoding of small RNA

The pre-PCR miRNA barcoding protocol is also similar to the Illumina Small RNA v1.5.

Sample Preparation Guide. The adapters used in the protocol were modified to include a barcode and to allow for Illumina index sequencing with the Illumina multiplexing index read sequencing primer. The complete protocol, including the adapter sequences, is available in Text S1.

### Preparation of dsDNA from S. cerevisiae mRNA

Starting with poly A+ enriched RNA from S. cerevisiae (Clontech 636312), dsDNA was prepared with the NEBNext mRNA Sample Prep Reagent Set 1 (New England Biolabs E6100). During this procedure, RNA was fragmented with a fragmentation buffer and subsequently purified with the Qiagen RNeasy Minelute kit. After second strand cDNA synthesis, the dsDNA was purified with a Zymo DNA Clean and concentrator-5.

### PALM DNA barcoding

The PALM DNA barcoding protocol is similar to the Illumina Genomic DNA Sample Preparation Guide. The adapters used in the protocol were modified to allow for PALM barcoding and Illumina index sequencing with the Illumina multiplexing index read sequencing primer. The complete protocol, including the adapter sequences, is available in Text S1. The main difference compared to the current Illumina Genomic DNA library protocol is the addition of the barcode to the library by ligation after PCR amplification. After ligation of the barcode, no further purification of the library is required. The library is quantified using analysis of area under the peaks with a BioAnalyzer 2100 (Agilent) to determine the correct loading concentration for subsequent sequencing.

### miRNA sequencing and data analysis

The pooled PALM and pre-PCR miRNA libraries were each sequenced in one lane on an Illumina Genome Analyzer IIx sequencer (40 bp single reads), using version 4 of cluster generation and sequencing kits. Sequencing of the pooled TruSeq miRNA libraries was done in one lane on an Illumina HiSeq 2000 sequencer (40 bp single reads), using version 4 of cluster generation and sequencing kits. Raw sequences were obtained from the Illumina GA Pipeline software CASAVA v1.7. The PALM barcoded sequences were demultiplexed using the Illumina pipeline and the pre-PCR barcodes using scripts written for this purpose. The pre-PCR barcodes cannot be demultiplexed using CASAVA because the pre-PCR barcode is not obtained with a separate read like the PALM and TruSeq barcode. The scripts allow for no mismatches in the barcode. Each barcode set was analyzed for small RNA using the Illumina pipeline add-on Flicker v2.7. Flicker trims the adaptor sequence from each read and does iterative alignment to the genome and to the miRNA database (miRBase v16) using the ELAND alignment strategy. The iterative alignment generates statistics of the number of reads aligning to the different classes of miRNA, as well as to individual miRNAs.

### DNA/mRNA sequencing and data analysis

The pooled *S. cerevisiae* mRNA libraries were sequenced in one lane on an Illumina HiSeq 2000 sequencer (40 bp single reads), using version 4 of cluster generation and sequencing kits. The Illumina GA Pipeline software CASAVA v1.7. was used to obtain the reads and to demultiplex the PALM and TruSeq barcoded sequences. Each barcode read set was aligned and annotated with CASAVA v1.7 using the *S. cerevisiae* S228C genome downloaded from the UCSC Genome website and the *S. cerevisiae* GTF exon and splice site annotation file downloaded from the Ensembl website. Reads that aligned to each exon and splice junction site were summed per gene.

### TruSeq small RNA and DNA barcoding and sequencing

For the TruSeq sample preparation, the Illumina TruSeq Small RNA Sample Prep Kit (RS-200–0012) and the Illumina TruSeq DNA Sample Prep Kit (FC-121–1001) were used.

## Results

### Yields and quantification of libraries

The PALM barcode ligation step produces several DNA products but only the main product, library products with the barcode adapters ligated to both ends, are able to form clusters and generate sequencing data. For miRNA libraries, this product has a size of approximately 170 bp. For mRNA/DNA libraries this product has a size that is 102 bp longer than the size selected product before the PCR step. The other DNA products present in the library cannot form clusters or be sequenced: Residual barcode adapters (∼32 bp) can bind to the Illumina flow cell with one end, but will not produce clusters because bridge amplification only occurs when both ends of the DNA strand bind to the flow cell. Barcode-adapter dimers (∼64 bp), can bind to the flow cell, but will not produce sequence because they lack a sequencing primer hybridization site. For this reason, no gel purification step is needed after the PALM barcode ligation step. When no final gel purification step is performed, quantification of the total quantity of DNA present in the library after barcode ligation would over-estimate the available material for optimal cluster generation and sequencing. Therefore, it is good practice to quantify the amounts of the desired products using an Agilent High Sensitivity DNA chip or an analogous gel- and microfluidics-based system to correctly load the flow cell.

For miRNA PALM barcoding, we optimized the yield of the Illumina small RNA library preparation protocol (version 1.5) for PALM barcoding by using higher reaction volumes for the RT-PCR step. No extra cycles were added to the PCR reaction. Starting from 1 µg of Human Brain Reference total RNA, the protocol yields 11.14±1.5 ng of gel purified PCR product. The PALM barcoding step worked well starting with between 2 and 20 ng of gel-purified, PCR-amplified miRNA library. After PALM barcoding and AMPure XP bead purification, the final yield (in ng) of library with barcodes ligated to both ends, is approximately the same as the amount of PCR-amplified miRNA library used to start the PALM barcoding reaction.

The mRNA/DNA PALM barcoding protocol is based on the Illumina Genomic DNA Sample Preparation Guide. Starting from 5 ng of dsDNA, the PALM protocol yields ∼200 ng PCR-amplified library (15 cycles) of which 100 ng was used in the PALM barcoding step. This generated >100 ng of library with barcodes ligated to both ends.

### Deep Sequencing Results of Human Brain Reference RNA

We performed multiplexed miRNA deep sequencing on Human Brain Reference RNA using libraries prepared with three different protocols: PALM barcoded (12 barcodes), pre-PCR barcoded (4 barcodes) and TruSeq barcoded (12 barcodes). Sequencing of the brain RNA yielded 23,685,700 Illumina GAIIx pass-filter reads for the PALM barcoded pool, 24,171,696 Illumina GAIIx reads for the pre-PCR barcoded pool and 35,495,446 Illumina HiSeq 2000 reads for the TruSeq pool. Of the pass-filter reads from the PALM, pre-PCR and TruSeq barcoded libraries, 88%, 92% and 97% contained the barcode sequence respectively. Representation of the differentially barcoded libraries within the flow cell lanes was uniform and more than 50% of all the sequences mapped to mature miRNAs (Table S1).

### Deep Sequencing Results of *S. cerevisiae* mRNA

We performed multiplexed mRNA deep sequencing on *S. cerevisiae* reference mRNA using libraries prepared with two different protocols: PALM barcoded (12 barcodes) and TruSeq barcoded (12 barcodes). Sequencing yielded 104,277,310 Illumina HiSeq 2000 pass-filter reads for the PALM barcoded pool and 115,419,701 Illumina HiSeq 2000 pass-filter reads for the TruSeq pool. Of the pass-filter reads from the PALM and TruSeq barcoded libraries, 94% and 97% contained the barcode sequence respectively. Representation of the differentially barcoded libraries within the flow cell lanes was uniform and more than 60% of all the sequences mapped to exons and splice junction sites (Table S2).

### Evaluation of bias for miRNA barcoding

**Figure 2:**
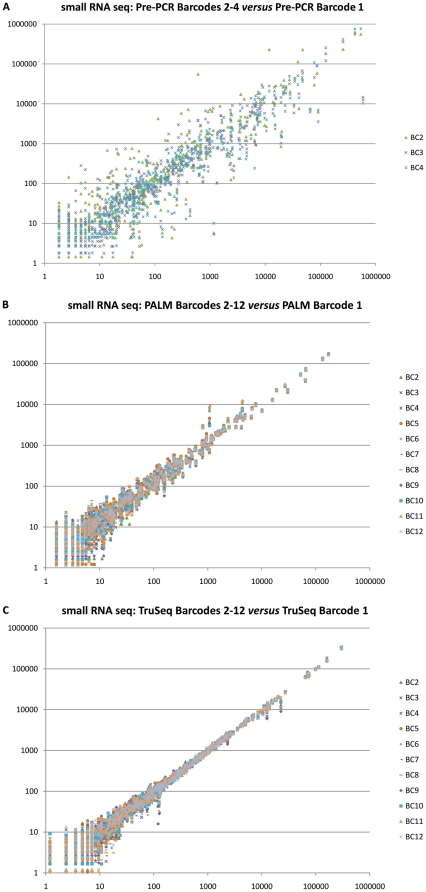
miRNA digital expression levels of all detected human brain reference sample miRNAs. (a) in the pre-PCR barcoded library 1 versus their expression in the 3 other pre-PCR barcoded libraries, (b) in the PALM barcoded library 1 versus their expression in the 11 other PALM barcoded libraries, (c) in the TruSeq barcoded library 1 versus their expression in the 11 other TruSeq barcoded libraries.

We calculated the expression of each miRNA as its number of read counts normalized by the total number of reads for each library. The scatter plots in Figure 2 shows a side-by-side comparison of the miRNA expression profiles of the human brain reference libraries, barcoded using either the pre-PCR (A), PALM barcoding protocol (B) and the TruSeq barcoding protocol (C). This comparison reveals a very low variability in the miRNA expression profiles of the PALM and TruSeq barcoded samples but not for the pre-PCR barcoded samples, which is confirmed using a linear regression analysis on the miRNA with at least 10 counts (Table S1) for one of the barcodes: Barcode 1 against the other barcodes gives an R^2^ = 0.8197±0.1217 for pre-PCR vs. R^2^ = 0.9930±0.0022 for PALM vs. R^2^ = 0.9977±0.0016 for TruSeq (See Table S3 for details). The bias introduced by the pre-PCR barcoding protocol precludes quantitative comparison of multiple samples using this strategy for multiplexing.

### Evaluation of bias for mRNA/dsDNA barcoding

**Figure 3:**
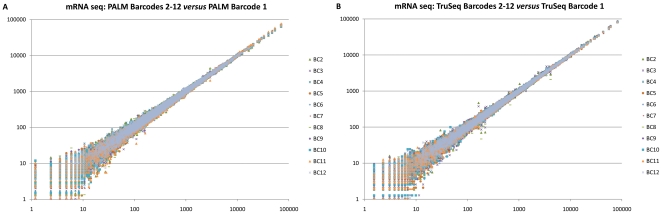
mRNA digital expression levels of all detected *S. cerevisiae* reference sample mRNAs. (a) in the PALM barcoded library 1 versus their expression in the 11 other PALM barcoded libraries, (b) in the TruSeq barcoded library 1 versus their expression in the 11 other TruSeq barcoded libraries.

We calculated the expression of each mRNA as its number of read counts normalized by the total number of reads for each library. The scatter plots in Figure 3 shows a side-by-side comparison of the mRNA expression profiles of the human brain reference libraries, barcoded using either the PALM barcoding protocol (A) and the TruSeq barcoding protocol (B). This comparison reveals a very low variability in the mRNA expression profiles of the PALM and TruSeq barcoded samples, which is confirmed using a linear regression analysis on the mRNA with at least 10 counts (Table S2) for one of the barcodes: Barcode 1 against the other barcodes gives an R^2^ = 0.9991±0.0005 for PALM vs. TruSeq R^2^ = 0.9996±0.0003 for TruSeq (See Table S3 for details).

## Discussion

The constantly increasing throughput of next generation sequencers opens the possibility for multiplexed sequencing of samples. For example, sequencing one miRNA sample in one flow cell lane on an Illumina GAIIx generates an order of magnitude more read data than required: There are currently only 1037 known human miRNAs, representing a maximum of 25 kb of reference sequence [10]. Current Illumina technology provides >50 million reads from 1 flow cell lane. Thus, even multiplexing 12 different miRNA samples in one lane results in >2 million reads per sample. This coverage is still enough to accurately quantify all but the low abundant miRNA present in these samples.

A commonly used technique for multiplexing samples for deep sequencing is to incorporate a known, sample-specific nucleotide sequence in the DNA fragments during library preparation [1]–[5]. This sample-specific sequence (barcode) is sequenced together with the rest of the fragment. PCR amplification of a pool of DNA molecules with different nucleotide compositions, especially near priming sites, can however result in quantitative bias because some DNA species are amplified more efficiently than others [6]–[9]. As we have shown here, introducing a barcode before the PCR step can result in a barcode-specific quantitative bias. Nonetheless, the currently published methods and commercial kits (i.e. Nugen and Bioo Scientific) introduce the barcode in the library before or during PCR-based library amplification. Unfortunately, none of these methods are provided with a quantitative analysis of the bias resulting from the use of barcodes. Thus, we reasoned that introduction of the barcode after library amplification would address this limitation by simply avoiding the problem and developed the PALM protocol. Illumina only recently introduced the TruSeq sample multiplexed sample preparation kits. The Illumina TruSeq small RNA strategy introduces the barcode during the PCR step using differentially barcoded primers, while the TruSeq DNA (or messenger RNA converted to double stranded DNA) strategy introduces the barcode before the PCR step by ligation of differentially barcoded adaptors. At the time of this publication, we are unaware of any published data demonstrating the impact of the TruSeq protocols on the bias created by the combination of barcoding and PCR. For this reason, we compared the PALM barcoding strategy with the TruSeq barcoding strategy.

Our results describe a detailed quantitative analysis of PCR and barcoding bias obtained using the PALM and the TruSeq barcoding protocol. The PALM protocol demonstrates a robust and efficient multiplexing method for miRNA and mRNA expression profiling that is free of barcode-induced PCR bias. In contrast, our results for the same miRNA samples profiled with our pre-PCR barcoding protocol demonstrate significant barcode-specific bias. This bias is quite extreme, as the digital expression of the same miRNAs shows up to 100-fold differences in read counts for the top 200 most abundantly expressed miRNAs. Both the TruSeq miRNA and mRNA/dsDNA barcoding protocols show no bias. In the TruSeq miRNA protocol, the strategy of introducing the barcode during the PCR step using differentially barcoded primers does not result in bias. The TruSeq protocol for mRNA/dsDNA which introduces the barcode before the PCR step, surprisingly also produces results with no bias. It is unclear why our pre-PCR protocol for small RNA produces biased results, while the TruSeq protocol for mRNA/dsDNA produces unbiased results. Compared to our pre-PCR small RNA protocol which places the barcode only 3 bp away from the miRNA insert, the TruSeq mRNA/dsDNA protocol places the barcode 34 bp away from the mRNA/dsDNA. Another difference is that the mRNA/dsDNA protocol contains no reverse transcriptase step after bacoding and works with a typical insert size of 250 bp, instead of the miRNA insert size of approx. 22 bp. Because of this, the barcode sequence might have less impact on the quantitative results after PCR.

There are multiple sources of bias that can be introduced during sample purification and library preparation including ligation bias, secondary structures, PCR-bias created by amplification of differentially barcoded miRNAs and amplification bias introduced on the surface of the flow cell [11]–[13]. The important point in the context of the present work is that PALM and TruSeq barcoding, in contrast to the pre-PCR barcoding protocol we used, gives consistent and reproducible results allowing multiplexing and meaningful comparisons of differential miRNA and mRNA expression without the need for technical replicates with different barcodes. In addition, PALM is a transparent and adaptable alternative to commercial strategies with a limited number of barcodes. It allows the user to modify the protocol and provides the flexibility to synthesize as many barcodes as needed in order to keep up with the ever-growing sequencing throughput.

## Supporting Information

Figure S1 **E-gel of a library after PCR amplification and before barcode ligation.** Typical Invitrogen 4% E-gel with 50 bp ladder of a Human Brain Reference RNA (Ambion) library after PCR amplification and before barcode ligation. The PCR product that needs to be purified from the gel is the band next to the 100 bp marker (second ladder band staring from the bottom of the picture). The bands closely above this PCR product should not be excised from the gel: Doing so lowers the percentage of mature miRNA sequences in the sequencing results.
(TIF)

Table S1 **Human Brain RNA sequence quality statistics.**
(DOCX)

Table S2 **S. cerevisiae mRNA sequence quality statistics.**
(DOCX)

Table S3 **Matrix of correlations between differentially barcoded samples.**
(DOCX)

Text S1 **Supplementary Materials and Methods.**
(DOCX)
