# Supplementary figures and images for: Quantitative Bias in Illumina TruSeq and a Novel Post Amplification Barcoding Strategy for Multiplexed DNA and Small RNA Deep Sequencing
Source: PLoS One. 2011 Oct 28;6(10):e26969. doi: 10.1371/journal.pone.0026969 (PMC3203936; doi:10.1371/journal.pone.0026969)

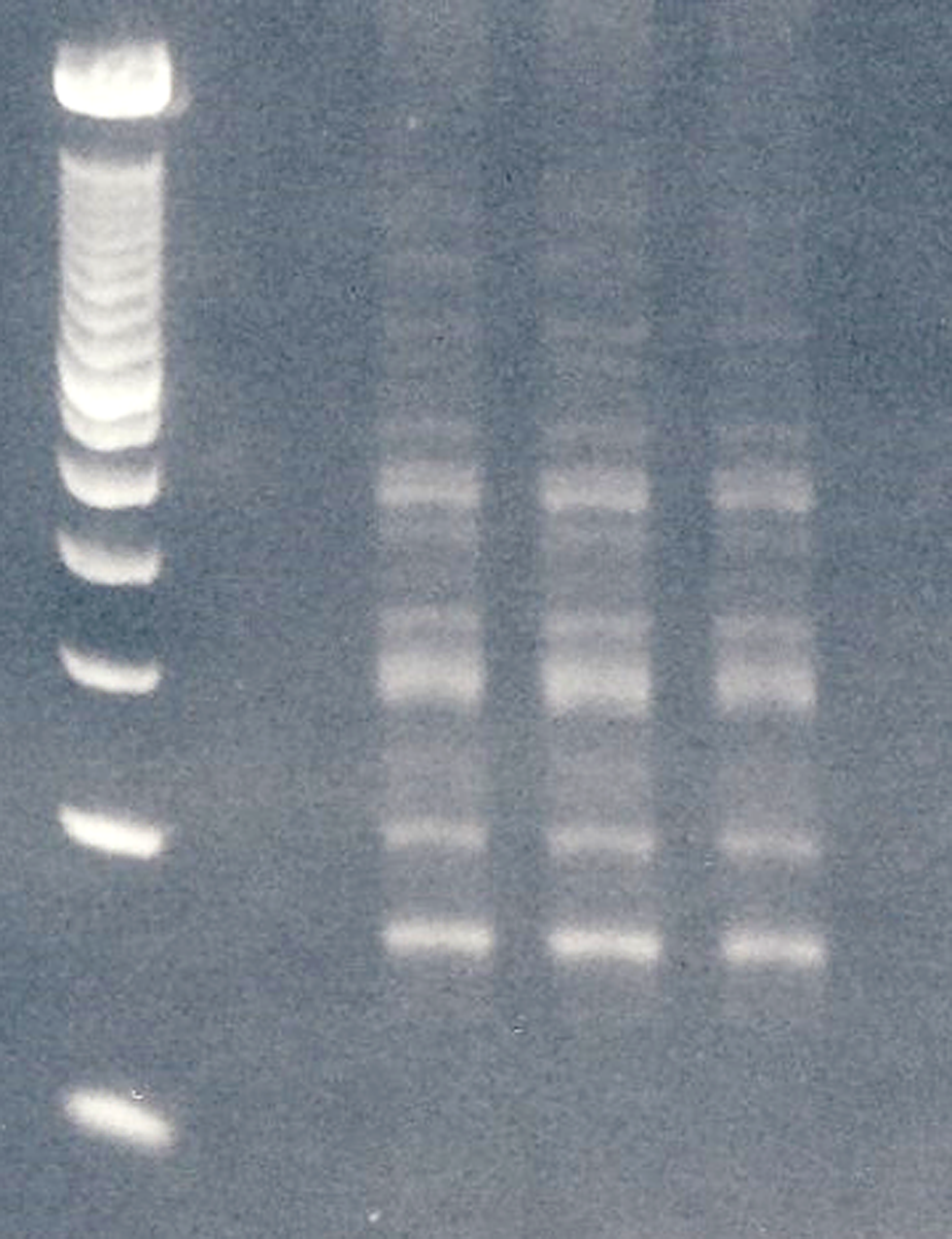

Supplement: Figure S1 — E-gel of a library after PCR amplification and before barcode ligation. Typical Invitrogen 4% E-gel with 50 bp ladder of a Human Brain Reference RNA (Ambion) library after PCR amplification and before barcode ligation. The PCR product that needs to be purified from the gel is the band next to the 100 bp marker (second ladder band staring from the bottom of the picture). The bands closely above this PCR product should not be excised from the gel: Doing so lowers the percentage of mature miRNA sequences in the sequencing results. (TIF) [file pone.0026969.s001.tif]
