# Supplementary material for: Quantitative Bias in Illumina TruSeq and a Novel Post Amplification Barcoding Strategy for Multiplexed DNA and Small RNA Deep Sequencing
Source: PLoS One. 2011 Oct 28;6(10):e26969. doi: 10.1371/journal.pone.0026969 (PMC3203936; doi:10.1371/journal.pone.0026969)
Supplement: Table S1 — Human Brain RNA sequence quality statistics. (DOCX) [file pone.0026969.s002.docx]

**Table S1:** Human Brain RNA sequence quality statistics

|  | **Number of reads (mean ± st. dev)**  **Dd** | **% (mean ± st. dev)** |
| --- | --- | --- |
| **12 PALM barcodes** |  |  |
| Total reads with barcodes | 20,598,984 |  |
| Ave reads per barcoded sample | 1,716,582 ± 372,711 |  |
| Unaligned – too short | 303,765 ± 126,807 | 17.58 ± 5.35 |
| Unaligned – no match | 123,341 ± 24,814 | 7.22 ± 0.53 |
| Aligned - mature | 904,344 ± 207,458 | 52.69 ± 4.74 |
| Identified miRNA | 491 ± 19 |  |
| miRNA with >10 counts | 325 |  |
| **4 pre-PCR barcodes** |  |  |
| Total reads with barcodes | 22,149,582 |  |
| Ave reads per barcoded sample | 5,537,396 ± 1,209,422 |  |
| Unaligned – too short | 359,639 ± 136,311 | 6.49 ±2.46 |
| Unaligned – no match | 261,967 ± 82,974 | 4.73 ±1.50 |
| Aligned - mature | 3,359,695 ± 698,009 | 60.67 ± 12.61 |
| Identified miRNA | 594 ± 4 |  |
| miRNA with >10 counts | 434 |  |
| **12 TruSeq barcodes** |  |  |
| Total reads with barcodes | 34,590,185 |  |
| Ave reads per barcoded sample | 2,882,515 ± 386,221 |  |
| Unaligned – too short | 63,515 ± 9,874 | 2.2 ± 0.34 |
| Unaligned – no match | 470,410 ± 56,970 | 16.32 ± 1.98 |
| Aligned - mature | 1,563,981 ± 211692 | 54.26 ± 7.34 |
| Identified miRNA | 581 ± 14 |  |
| miRNA with >10 counts | 402 |  |

Alignment statistics were generated by Flicker version 2.7. The pool of 12 PALM barcodes and the pool of 4 Pre-PCR barcodes were each sequenced in one Illumina GAIIx flow cell lane. The pool of 12 TruSeq barcodes was sequenced in one Illumina HiSeq 2000 lane. The table also shows the number of identified miRNA in each barcoded sample and the number of miRNA with at least 10 counts in at least one of the barcoded samples.
