# Supplementary material for: Quantitative Bias in Illumina TruSeq and a Novel Post Amplification Barcoding Strategy for Multiplexed DNA and Small RNA Deep Sequencing
Source: PLoS One. 2011 Oct 28;6(10):e26969. doi: 10.1371/journal.pone.0026969 (PMC3203936; doi:10.1371/journal.pone.0026969)
Supplement: Table S2 — S. cerevisiae mRNA sequence quality statistics. (DOCX) [file pone.0026969.s003.docx]

**Table S2**: S. cerevisiae mRNA sequence quality statistics

|  | **Number of reads (mean ± st. dev)**  **dd** | **% (mean ± st. dev)** |
| --- | --- | --- |
| **12 PALM barcodes** |  |  |
| Total reads with barcodes | 98,108,551 |  |
| Ave reads per barcoded sample | 8,175,712 ± 2,018,971 |  |
| Aligned | 5,287,979 ± 1,315,639 | 0,64 ± 0,16 |
| Unaligned | 2,887,734 ± 703,653 | 0,35 ± 0,09 |
| Identified mRNA | 6482 ± 24 |  |
| miRNA with >10 counts | 6208 |  |
| **12 TruSeq barcodes** |  |  |
| Total reads with barcodes | 112,036,259 |  |
| Ave reads per barcoded sample | 9,336,354 ± 1,405,129 |  |
| Aligned | 6,120,073 ± 929,944 | 0,66 ± 0,10 |
| Unaligned | 3,216,282 ± 477,117 | 0,34 ± 0,05 |
| Identified mRNA | 6496 ± 14 |  |
| miRNA with >10 counts | 6230 |  |

Alignment statistics were generated with CASAVA v1.7. The pool of 12 PALM barcodes and the pool of 12 TruSeq barcodes were each sequenced in one Illumina HiSeq 2000 lane. The table also shows the number of identified mRNA for each barcoded sample and the number of miRNA with at least 10 counts in at least one of the barcoded samples.
