# Supplementary material for: Quantitative Bias in Illumina TruSeq and a Novel Post Amplification Barcoding Strategy for Multiplexed DNA and Small RNA Deep Sequencing
Source: PLoS One. 2011 Oct 28;6(10):e26969. doi: 10.1371/journal.pone.0026969 (PMC3203936; doi:10.1371/journal.pone.0026969)
Supplement: Table S3 — Matrix of correlations between differentially barcoded samples. (DOCX) [file pone.0026969.s004.docx]

**Table S3**: Matrix of correlations between differentially barcoded samples

| **miRNA: Pre-PCR barcoding** | | | | |  |  |  |  |  |  |  |  |
| --- | --- | --- | --- | --- | --- | --- | --- | --- | --- | --- | --- | --- |
|  | BC 1 | BC 2 | BC 3 | BC 4 |  |  |  |  |  |  |  |  |
| BC 1 | 1,0000 | 0,7334 | 0,7677 | 0,7774 |  |  |  |  |  |  |  |  |
| BC 2 | 0,7334 | 1,0000 | 0,8859 | 0,9363 |  |  |  |  |  |  |  |  |
| BC 3 | 0,7677 | 0,8859 | 1,0000 | 0,9479 |  |  |  |  |  |  |  |  |
| BC 4 | 0,7774 | 0,9363 | 0,9479 | 1,0000 |  |  |  |  |  |  |  |  |
| **miRNA: PALM barcoding** | | | | |  |  |  |  |  |  |  |  |
|  | BC 1 | BC 2 | BC 3 | BC 4 | BC 5 | BC 6 | BC 7 | BC 8 | BC 9 | BC 10 | BC 11 | BC 12 |
| BC 1 | 1,0000 | 0,9923 | 0,9920 | 0,9919 | 0,9928 | 0,9924 | 0,9919 | 0,9929 | 0,9930 | 0,9929 | 0,9921 | 0,9922 |
| BC 2 | 0,9923 | 1,0000 | 0,9982 | 0,9986 | 0,9993 | 0,9995 | 0,9976 | 0,9994 | 0,9989 | 0,9983 | 0,9990 | 0,9968 |
| BC 3 | 0,9920 | 0,9982 | 1,0000 | 0,9975 | 0,9995 | 0,9988 | 0,9992 | 0,9989 | 0,9997 | 0,9992 | 0,9983 | 0,9986 |
| BC 4 | 0,9919 | 0,9986 | 0,9975 | 1,0000 | 0,9981 | 0,9990 | 0,9984 | 0,9986 | 0,9986 | 0,9989 | 0,9994 | 0,9982 |
| BC 5 | 0,9928 | 0,9993 | 0,9995 | 0,9981 | 1,0000 | 0,9995 | 0,9988 | 0,9996 | 0,9996 | 0,9989 | 0,9989 | 0,9978 |
| BC 6 | 0,9924 | 0,9995 | 0,9988 | 0,9990 | 0,9995 | 1,0000 | 0,9990 | 0,9999 | 0,9992 | 0,9986 | 0,9997 | 0,9975 |
| BC 7 | 0,9919 | 0,9976 | 0,9992 | 0,9984 | 0,9988 | 0,9990 | 1,0000 | 0,9990 | 0,9993 | 0,9992 | 0,9992 | 0,9991 |
| BC 8 | 0,9929 | 0,9994 | 0,9989 | 0,9986 | 0,9996 | 0,9999 | 0,9990 | 1,0000 | 0,9993 | 0,9986 | 0,9996 | 0,9975 |
| BC 9 | 0,9930 | 0,9989 | 0,9997 | 0,9986 | 0,9996 | 0,9992 | 0,9993 | 0,9993 | 1,0000 | 0,9998 | 0,9990 | 0,9991 |
| BC 10 | 0,9929 | 0,9983 | 0,9992 | 0,9989 | 0,9989 | 0,9986 | 0,9992 | 0,9986 | 0,9998 | 1,0000 | 0,9988 | 0,9997 |
| BC 11 | 0,9921 | 0,9990 | 0,9983 | 0,9994 | 0,9989 | 0,9997 | 0,9992 | 0,9996 | 0,9990 | 0,9988 | 1,0000 | 0,9980 |
| BC 12 | 0,9922 | 0,9968 | 0,9986 | 0,9982 | 0,9978 | 0,9975 | 0,9991 | 0,9975 | 0,9991 | 0,9997 | 0,9980 | 1,0000 |
| **miRNA: TruSeq barcoding** | | | | |  |  |  |  |  |  |  |  |
|  | BC 1 | BC 2 | BC 3 | BC 4 | BC 5 | BC 6 | BC 7 | BC 8 | BC 9 | BC 10 | BC 11 | BC 12 |
| BC 1 | 1,0000 | 0,9988 | 0,9955 | 0,9977 | 0,9988 | 0,9991 | 0,9944 | 0,9975 | 0,9972 | 0,9970 | 0,9990 | 0,9976 |
| BC 2 | 0,9988 | 1,0000 | 0,9971 | 0,9987 | 0,9996 | 0,9995 | 0,9963 | 0,9982 | 0,9991 | 0,9984 | 0,9995 | 0,9993 |
| BC 3 | 0,9955 | 0,9971 | 1,0000 | 0,9956 | 0,9983 | 0,9984 | 0,9996 | 0,9992 | 0,9992 | 0,9995 | 0,9975 | 0,9984 |
| BC 4 | 0,9977 | 0,9987 | 0,9956 | 1,0000 | 0,9980 | 0,9982 | 0,9957 | 0,9970 | 0,9972 | 0,9976 | 0,9983 | 0,9986 |
| BC 5 | 0,9988 | 0,9996 | 0,9983 | 0,9980 | 1,0000 | 0,9998 | 0,9972 | 0,9990 | 0,9995 | 0,9989 | 0,9992 | 0,9994 |
| BC 6 | 0,9991 | 0,9995 | 0,9984 | 0,9982 | 0,9998 | 1,0000 | 0,9977 | 0,9994 | 0,9993 | 0,9991 | 0,9996 | 0,9993 |
| BC 7 | 0,9944 | 0,9963 | 0,9996 | 0,9957 | 0,9972 | 0,9977 | 1,0000 | 0,9988 | 0,9984 | 0,9992 | 0,9969 | 0,9980 |
| BC 8 | 0,9975 | 0,9982 | 0,9992 | 0,9970 | 0,9990 | 0,9994 | 0,9988 | 1,0000 | 0,9990 | 0,9995 | 0,9989 | 0,9991 |
| BC 9 | 0,9972 | 0,9991 | 0,9992 | 0,9972 | 0,9995 | 0,9993 | 0,9984 | 0,9990 | 1,0000 | 0,9994 | 0,9988 | 0,9994 |
| BC 10 | 0,9970 | 0,9984 | 0,9995 | 0,9976 | 0,9989 | 0,9991 | 0,9992 | 0,9995 | 0,9994 | 1,0000 | 0,9988 | 0,9991 |
| BC 11 | 0,9990 | 0,9995 | 0,9975 | 0,9983 | 0,9992 | 0,9996 | 0,9969 | 0,9989 | 0,9988 | 0,9988 | 1,0000 | 0,9990 |
| BC 12 | 0,9976 | 0,9993 | 0,9984 | 0,9986 | 0,9994 | 0,9993 | 0,9980 | 0,9991 | 0,9994 | 0,9991 | 0,9990 | 1,0000 |
| **mRNA / dsDNA: PALM barcoding** | | | |  |  |  |  |  |  |  |  |  |
|  | BC 1 | BC 2 | BC 3 | BC 4 | BC 5 | BC 6 | BC 7 | BC 8 | BC 9 | BC 10 | BC 11 | BC 12 |
| BC 1 | 1,0000 | 0,9982 | 0,9995 | 0,9985 | 0,9991 | 0,9989 | 0,9994 | 0,9985 | 0,9993 | 0,9996 | 0,9989 | 0,9988 |
| BC 2 | 0,9982 | 1,0000 | 0,9978 | 0,9995 | 0,9994 | 0,9995 | 0,9969 | 0,9997 | 0,9992 | 0,9991 | 0,9955 | 0,9995 |
| BC 3 | 0,9995 | 0,9978 | 1,0000 | 0,9987 | 0,9989 | 0,9983 | 0,9988 | 0,9981 | 0,9988 | 0,9993 | 0,9991 | 0,9986 |
| BC 4 | 0,9985 | 0,9995 | 0,9987 | 1,0000 | 0,9996 | 0,9993 | 0,9971 | 0,9995 | 0,9991 | 0,9993 | 0,9965 | 0,9996 |
| BC 5 | 0,9991 | 0,9994 | 0,9989 | 0,9996 | 1,0000 | 0,9996 | 0,9981 | 0,9995 | 0,9995 | 0,9997 | 0,9973 | 0,9996 |
| BC 6 | 0,9989 | 0,9995 | 0,9983 | 0,9993 | 0,9996 | 1,0000 | 0,9981 | 0,9996 | 0,9997 | 0,9995 | 0,9966 | 0,9996 |
| BC 7 | 0,9994 | 0,9969 | 0,9988 | 0,9971 | 0,9981 | 0,9981 | 1,0000 | 0,9975 | 0,9987 | 0,9988 | 0,9991 | 0,9978 |
| BC 8 | 0,9985 | 0,9997 | 0,9981 | 0,9995 | 0,9995 | 0,9996 | 0,9975 | 1,0000 | 0,9993 | 0,9992 | 0,9961 | 0,9996 |
| BC 9 | 0,9993 | 0,9992 | 0,9988 | 0,9991 | 0,9995 | 0,9997 | 0,9987 | 0,9993 | 1,0000 | 0,9997 | 0,9974 | 0,9994 |
| BC 10 | 0,9996 | 0,9991 | 0,9993 | 0,9993 | 0,9997 | 0,9995 | 0,9988 | 0,9992 | 0,9997 | 1,0000 | 0,9981 | 0,9995 |
| BC 11 | 0,9989 | 0,9955 | 0,9991 | 0,9965 | 0,9973 | 0,9966 | 0,9991 | 0,9961 | 0,9974 | 0,9981 | 1,0000 | 0,9968 |
| BC 12 | 0,9988 | 0,9995 | 0,9986 | 0,9996 | 0,9996 | 0,9996 | 0,9978 | 0,9996 | 0,9994 | 0,9995 | 0,9968 | 1,0000 |
| **mRNA / dsDNA: TruSeq barcoding** | | | |  |  |  |  |  |  |  |  |  |
|  |  |  |  |  |  |  |  |  |  |  |  |  |
|  | BC 1 | BC 2 | BC 3 | BC 4 | BC 5 | BC 6 | BC 7 | BC 8 | BC 9 | BC 10 | BC 11 | BC 12 |
| BC 1 | 1,0000 | 0,9994 | 0,9989 | 0,9998 | 0,9997 | 0,9996 | 0,9991 | 0,9994 | 0,9997 | 0,9997 | 0,9997 | 0,9996 |
| BC 2 | 0,9994 | 1,0000 | 0,9994 | 0,9992 | 0,9991 | 0,9996 | 0,9989 | 0,9992 | 0,9992 | 0,9993 | 0,9990 | 0,9994 |
| BC 3 | 0,9989 | 0,9994 | 1,0000 | 0,9985 | 0,9983 | 0,9989 | 0,9981 | 0,9981 | 0,9985 | 0,9993 | 0,9987 | 0,9994 |
| BC 4 | 0,9998 | 0,9992 | 0,9985 | 1,0000 | 0,9998 | 0,9996 | 0,9991 | 0,9995 | 0,9998 | 0,9994 | 0,9995 | 0,9993 |
| BC 5 | 0,9997 | 0,9991 | 0,9983 | 0,9998 | 1,0000 | 0,9996 | 0,9992 | 0,9995 | 0,9998 | 0,9992 | 0,9993 | 0,9991 |
| BC 6 | 0,9996 | 0,9996 | 0,9989 | 0,9996 | 0,9996 | 1,0000 | 0,9990 | 0,9995 | 0,9996 | 0,9993 | 0,9991 | 0,9992 |
| BC 7 | 0,9991 | 0,9989 | 0,9981 | 0,9991 | 0,9992 | 0,9990 | 1,0000 | 0,9994 | 0,9991 | 0,9987 | 0,9988 | 0,9988 |
| BC 8 | 0,9994 | 0,9992 | 0,9981 | 0,9995 | 0,9995 | 0,9995 | 0,9994 | 1,0000 | 0,9995 | 0,9989 | 0,9991 | 0,9989 |
| BC 9 | 0,9997 | 0,9992 | 0,9985 | 0,9998 | 0,9998 | 0,9996 | 0,9991 | 0,9995 | 1,0000 | 0,9994 | 0,9994 | 0,9993 |
| BC 10 | 0,9997 | 0,9993 | 0,9993 | 0,9994 | 0,9992 | 0,9993 | 0,9987 | 0,9989 | 0,9994 | 1,0000 | 0,9996 | 0,9998 |
| BC 11 | 0,9997 | 0,9990 | 0,9987 | 0,9995 | 0,9993 | 0,9991 | 0,9988 | 0,9991 | 0,9994 | 0,9996 | 1,0000 | 0,9996 |
| BC 12 | 0,9996 | 0,9994 | 0,9994 | 0,9993 | 0,9991 | 0,9992 | 0,9988 | 0,9989 | 0,9993 | 0,9998 | 0,9996 | 1,0000 |

Only miRNA and mRNA with at least 10 counts in at least one of the barcoded samples were considered
