# Supplementary material for: Quantitative Bias in Illumina TruSeq and a Novel Post Amplification Barcoding Strategy for Multiplexed DNA and Small RNA Deep Sequencing
Source: PLoS One. 2011 Oct 28;6(10):e26969. doi: 10.1371/journal.pone.0026969 (PMC3203936; doi:10.1371/journal.pone.0026969)
Supplement: Text S1 — Supplementary Materials and Methods. (DOCX) [file pone.0026969.s005.docx]

**Supporting Text S1: Supplementary Materials and Methods**

**Quantitative Bias in Illumina TruSeq and a Novel Post Amplification Barcoding Strategy for Multiplexed DNA and small RNA Deep Sequencing**

**Filip Van Nieuwerburgh, Sandra Soetaert, Katie Podshivalova, Eileen Ay-Lin Wang, Lana Schaffer, Dieter Deforce, Daniel R Salomon, Steven R Head, Phillip T. Ordoukhanian**

**Table of Contents**

**Supplementary Materials and Methods**

1) PALM barcoding of small RNA

1. Materials
2. Methods

2) Pre-PCR barcoding of small RNA

1. Materials
2. Methods

3) PALM barcoding of DNA

1. Materials
2. Methods

**Supplementary Materials and Methods**

**PALM Barcoding of small RNA**

**Materials:** All oligonucleotides were ordered HPLC purified from Integrated DNA Technologies Inc. (IDT). Enzymes were obtained from Invitrogen, Life Technologies, Inc., New England Biolabs, and Enzymatics Inc.

- PALM Adapters

5X PALM miRNA 3′adapter (10 µM) 5′ **rApp**-AGATCGGAAGAGCACACGTCT(**C3spacer**)

PALM miRNA 5′adapter (5 µM) 5′ GUUCAGAGUUCUACAGUCCGACGAUC

- 5X PALM Reverse Transcription primer

5X PALM RT primer (100 µM) 5′ GTGACTGGAGTTCAGACGTGTGCTCTTCCGA

- PALM PCR primers

PALM miRNA PCR primer 1 (25 µM) 5′ AATGATACGGCGACCACCGACAGGTTCAGAGTTCTACAGTCCGA

PALM miRNA PCR primer 2 (25 µM) 5′ **P**-GTGACTGGAGTTCAGACGTGTGCTCTTCCGA

- PALM Barcodes (50 µM final concentration in annealed solution 50mM NaCl/10mM Tris pH 7.5)

barcode 1-P 5′ **P**-G-ATCACG-ATCTCGTATGCCGTCTTCTGCTTG/3AmMO/

barcode 1-T 5′ CAAGCAGAAGACGGCATACGAGATCGTGATC*****T

barcode 2-P 5′ **P**-G-CGATGT-ATCTCGTATGCCGTCTTCTGCTTG/3AmMO/

barcode 2-T 5′ CAAGCAGAAGACGGCATACGAGATACATCGC*****T

barcode 3-P 5′ **P**-G-TTAGGC-ATCTCGTATGCCGTCTTCTGCTTG/3AmMO/

barcode 3-T 5′ CAAGCAGAAGACGGCATACGAGATGCCTAAC*****T

barcode 4-P 5′ **P**-G-TGACCA-ATCTCGTATGCCGTCTTCTGCTTG/3AmMO/

barcode 4-T 5′ CAAGCAGAAGACGGCATACGAGATTGGTCAC*****T

barcode 5-P 5′ **P**-G-ACAGTG-ATCTCGTATGCCGTCTTCTGCTTG/3AmMO/

barcode 5-T 5′ CAAGCAGAAGACGGCATACGAGATCACTGTC*****T

barcode 6-P 5′ **P**-G-GCCAAT-ATCTCGTATGCCGTCTTCTGCTTG/3AmMO/

barcode 6-T 5′ CAAGCAGAAGACGGCATACGAGATATTGGCC*****T

barcode 7-P 5′ **P**-G-CAGATC-ATCTCGTATGCCGTCTTCTGCTTG/3AmMO/

barcode 7-T 5′ CAAGCAGAAGACGGCATACGAGATGATCTGC*****T

barcode 8-P 5′ **P**-G-ACTTGA-ATCTCGTATGCCGTCTTCTGCTTG/3AmMO/

barcode 8-T 5′ CAAGCAGAAGACGGCATACGAGATTCAAGTC*****T

barcode 9-P 5′ **P**-G-GATCAG-ATCTCGTATGCCGTCTTCTGCTTG/3AmMO/

barcode 9-T 5′ CAAGCAGAAGACGGCATACGAGATCTGATCC*****T

barcode 10-P 5′ **P**-G-TAGCTT-ATCTCGTATGCCGTCTTCTGCTTG/3AmMO/

barcode 10-T 5′ CAAGCAGAAGACGGCATACGAGATAAGCTAC*****T

barcode 11-P 5′ **P**-G-GGCTAC-ATCTCGTATGCCGTCTTCTGCTTG/3AmMO/

barcode 11-T 5′ CAAGCAGAAGACGGCATACGAGATGTAGCCC*****T

barcode 12-P 5′ **P**-G-CTTGTA-ATCTCGTATGCCGTCTTCTGCTTG/3AmMO/

barcode 12-T 5′ CAAGCAGAAGACGGCATACGAGATTACAAGC*****T

(**P =** 5′**phosphate, 3AmMO = 3**′**amine, and * = phosphorothioate linkage)**

- T4 RNA Ligase 2, truncated; T4 RNA Ligase; Finnzymes 2X Phusion HF master mix; and Taq DNA polymerase (New England Biolabs)
- RNaseOUT, SuperScript™ II Reverse Transcriptase, Qubit Fluorometer, dsDNA High Sensitivity assay, Magnetic Particle Concentrator, E-Gel EX 4% Agarose Gel, TrackIt 50 bp ladder, and 10 mM ATP (Invitrogen, Life Technologies, Inc.)
- Agarose Dissolving Buffer (ADB) and DNA Clean and concentrator -5 and -25 kits (Zymo Research Co.)
- T4 DNA Ligase (Rapid), using 2X Rapid Ligation Buffer (Enzymatics, Inc.)
- Molecular Biology Grade, DNase- and RNase-free MgCl_2_ (Sigma-Aldrich, Co.)
- 2100 Bioanalyzer and High Sensitivity DNA kit (Agilent Technologies)
- Agencourt AMPure XP magnetic beads (Beckman Coulter, Inc.)
- Dark Reader® transilluminator (Clare Chemical Research, Inc.)

**Methods:**

1. **Ligation of 3′- and 5′- adapters to total RNA**

1 µg of total RNA sample was added to 10 pmol of PALM miRNA 3**′** adapter in a final volume of 6 µL. The mixture was incubated at 70°C for 2 minutes, and then transferred to ice. Subsequently, the following reagents were added to the mixture: 1 μL 10X T4 RNA Ligase 2 truncated reaction buffer; 0.8 μL 100 mM MgCl2; 1.5 μL T4 RNA Ligase 2 truncated; and 0.5 μL RNaseOUT, and the reaction was incubated at 22°C for 1 hour. Just prior to this reaction finishing, the PALM miRNA 5**′** adapter was denatured by heating it at 70°C for 2 minutes and transferring it to ice. Then, the following reagents were added to the reaction mixture: 1 μL of 10 mM ATP; 1 μL PALM miRNA 5′ Adapter; and 1 μL T4 RNA Ligase. The reaction was incubated at 20°C for 1 hour and then transferred to ice.

1. **Reverse transcription of adapter ligated products.**

12 μL of the above RNA Ligation reaction was then taken and added directly to 60 pmol of PALM RT primer in a final volume of 15 μL. The mixture was then heated to 70°C for 2 minutes, and then transferred to ice. In a separate, nuclease-free PCR tube the following reagents were premixed: 6 μL 5X First Strand Buffer; 1.5 μL 12.5 mM dNTP mix; 3 μL 100 mM DTT; and 1.5 μL RNaseOUT. The 15 μL of Ligation products and PALM RT primer were then combined with the 12 μL of reagents in the PCR tube. The mixture was heated to 48°C for 3 minutes, and then 3 μL SuperScript™ II was added. The reaction was then incubated at 44°C for 1 hour, and then transferred to ice.

1. **PCR Amplification.**

In a separate microfuge tube the following reagents were combined and placed on ice: 50 μL 2X phusion master mix; 2 uL PALM miRNA PCR primer 1; 2 uL PALM miRNA PCR primer 2; 16 μL nuclease-free purified water. This mixture of reagents was then added to the 30 μL from the reverse transcription reaction in a PCR tube. The mixture was then placed in a thermocycler using the following program: 1 initial hold, 98ºC, 30 seconds; cycled 12 times, 98ºC, 10 seconds; 60ºC, 30 seconds; and 72ºC, 15 seconds; 1 final hold, 72ºC, 10 minutes.

After the PCR reaction was complete, the PCR products were purified using a Zymo-25 DNA column clean up protocol and eluted from the column in 38.5 µL nuclease-free purified water.

1. **3′ A-tailing of PCR products.**

The eluted PCR products were mixed with 5 µl 10X Taq buffer, 5 μL 10 mM dATP and 1.5 µL Taq polymerase. The reaction was then incubated at 72°C for 30 minutes. The A-tailed PCR products were then isolated from the reaction using a Zymo-25 DNA column clean up protocol and eluted from the column in 60 µL nuclease-free purified water.

1. **Purification of PCR products.**

The desired PCR products were purified by loading onto a 4% Agarose E-gel, 20 μL per lane (3 lanes per sample). 5 μL of TrackIT 50bp Ladder was loaded into a single lane and used as a DNA size marker. The gel was electrophoresed for approximately 30 minutes, until the desired bands (at approximately 100bp ± 10bp, see Supplementary Figure 1), were at the bottom of the gel. The gel was imaged using a Dark Reader® Transilluminator and the desired bands were excised using a clean razor blade. Approximately, 4 volumes of ADB buffer were added to the gel pieces and the mixture was incubated for 5-10 minutes at 37 ºC, while mixing several times during the period. PCR products were isolated from the mixture using a Zymo-5 DNA column clean up protocol. The desired PCR products were eluted from the column with 21 μL nuclease-free, purified water. 1 µL of the eluted DNA products was used for quantification with a Qubit Fluorometer.

1. **Ligation of PALM barcodes to PCR products.**

The amount of material going into ligation reaction varied but worked well at 2-20 ng. The eluted PCR products were mixed with 25 pmol of annealed PALM barcode in a final volume of 20 μL and added to 25 μL of 2X Rapid Ligation buffer with 5 μL T4 DNA Ligase (Rapid) and incubated at 20°C for 30 minutes. 1 µl of 0.5 M EDTA was added to stop the ligation reaction.

1. **AMPure XP magnetic bead isolation of DNA products.**

After the ligation reaction, 90 μl of AMPure XP beads was added, mixed, and allowed to incubate at room temperature for 5 minutes. Using a Magnetic Particle Concentrator (MPC), the beads were pelleted against the wall of the tube. The tube will remained in the MCP during all subsequent wash steps. The supernatant was removed, and the beads were washed twice with 500 μL of 70% ethanol (allowed to sit for 1 minute each time during wash). The AMPure XP beads were allowed to air dry for approximately 5 minutes. Then the tube was removed from the MPC and 30 μL of TE was added and vortexed to re-suspend the beads. After a 2 minute wait step, the tube was placed back in the MPC to pellet the beads against the wall of the tube and the eluate was removed and transferred to a fresh microfuge tube.

1. **Agilent High Sensitivity DNA chip analysis and Illumina flow cell loading.**

1 µl of the eluted DNA library was run on an Agilent High Sensitivity DNA chip to determine size and concentration of the desired barcoded products (at approximately 145bp and 180bp). The concentration of the desired products was determined from the Agilent 2100 Bioanalyzer electropherogram and used to prepare an equimolar pool of 9 pM solution for cluster generation on an Illumina flow cell.

**Pre-PCR Barcoding of small RNA**

**Materials:** All oligonucleotides were ordered HPLC purified from Integrated DNA Technologies Inc. (IDT). Enzymes were obtained from Invitrogen, Life Technologies, Inc., New England Biolabs, and Enzymatics Inc.

- Adapters

5X miRNA 3′adapter (10 µM) 5′ **rApp**-ATCTCGTATGCCGTCTTCTGCTTG(**ddC**)

miRNA 5′adapter (5 µM) 5′ GUUCAGAGUUCUACAGUCCGACGAUC

Note: Bases in red are the barcode sequences. The 4 barcodes used in this study were:

BC1 5′ GUUCAGAGUUCUACAGUCCGACGAUC GACG AUC

BC2 5′ GUUCAGAGUUCUACAGUCCGACGAUC AAGA AUC

BC3 5′ GUUCAGAGUUCUACAGUCCGACGAUC ACUC AUC

BC4 5′ GUUCAGAGUUCUACAGUCCGACGAUC CGCA AUC

- 5X Reverse Transcription primer

5X miRNA RT primer (100 µM) 5′ CAAGCAGAAGACGGCATACGA

- PCR primers

miRNA PCR primer 1 (25 µM) 5′ CAAGCAGAAGACGGCATACGA

miRNA PCR primer 2 (25 µM) 5′ AATGATACGGCGACCACCGACAGGTTCAGAGTTCTACAGTCCGA

- T4 RNA Ligase 2, truncated; T4 RNA Ligase; and Finnzymes 2X Phusion HF master mix (New England Biolabs)
- RNaseOUT, SuperScript™ II Reverse Transcriptase, Qubit Fluorometer, dsDNA High Sensitivity assay, Magnetic Particle Concentrator, E-Gel EX 4% Agarose Gel, TrackIt 50 bp ladder and 10 mM ATP (Invitrogen, Life Technologies, Inc.)
- Agarose Dissolving Buffer (ADB) and DNA Clean and concentrator -5 and -25 kits (Zymo Research Co.)
- Molecular Biology Grade, DNase- and RNase-free MgCl_2_ (Sigma-Aldrich, Co.)
- 2100 Bioanalyzer and High Sensitivity DNA kit (Agilent Technologies)
- Agencourt AMPure XP magnetic beads (Beckman Coulter, Inc.)
- Dark Reader® transilluminator (Clare Chemical Research, Inc.)

**Methods:**

1. **Ligation of 3′- and 5′- adapters to total RNA**

1 µg of total RNA sample was added to 10 pmol of miRNA 3′ adapter in a final volume of 6 µL. The mixture was incubated at 70°C for 2 minutes, and then transferred to ice. Subsequently, the following reagents were added to the mixture: 1 μL 10X T4 RNA Ligase 2 truncated reaction buffer; 0.8 μL 100 mM MgCl2; 1.5 μL T4 RNA Ligase 2 truncated; and 0.5 μL RNaseOUT, and the reaction was incubated at 22°C for 1 hour. Just prior to this reaction finishing, the miRNA 5′ adapter was denatured by heating it at 70°C for 2 minutes and transferring it to ice. Then, the following reagents were added to the reaction mixture: 1 μL of 10 mM ATP; 1 μL miRNA 5′ Adapter; and 1 μL T4 RNA Ligase. The reaction was incubated at 20°C for 1 hour and then transferred to ice.

1. **Reverse transcription of adapter ligated products.**

8 μL of the above RNA Ligation reaction was added directly to 40 pmol of miRNA RT primer in a final volume of 10 μL. The mixture was heated to 70°C for 2 minutes and then transferred to ice. In a separate, nuclease-free PCR tube the following reagents were premixed: 4 μL 5X First Strand Buffer; 1 μL 12.5 mM dNTP mix; 2 μL 100 mM DTT; and 1 μL RNaseOUT. The 10 μL of ligation products and miRNA RT primer were then combined with the 8 μL of reagents in the PCR tube. The mixture was heated to 48°C for 3 minutes and then 2 μL SuperScript™ II was added. The reaction was then incubated at 44°C for 1 hour and then transferred to ice.

1. **PCR Amplification.**

In a separate microfuge tube the following reagents were combined and placed on ice: 25 μL 2X phusion master mix; 1 uL miRNA PCR primer 1; 1 uL miRNA PCR primer 2 and 3 μL nuclease-free purified water. This mixture of reagents was then added to the 20 μL from the reverse transcription reaction in the PCR tube. The mixture was then placed in a thermocycler using the following program: 1 initial hold, 98ºC, 30 seconds; cycled 12 times, 98ºC, 10 seconds; 60ºC, 30 seconds; and 72ºC, 15 seconds; 1 final hold, 72ºC, 10 minutes.

The PCR products were then isolated from the reaction using a Zymo-25 DNA column clean up protocol and eluted from the column in 60 µL nuclease-free purified water.

1. **Purification of PCR products.**

The desired PCR products were purified by loading onto a 4% Agarose E-gel, 20 μL per lane (3 lanes per sample). 5 μL of TrackIT 50bp Ladder was loaded into a single lane and used as a DNA size marker. The gel was electrophoresed for approximately 30 minutes, until the desired bands (at approximately 100bp ± 10bp, see Supplementary Figure 1), were at the bottom of the gel. The gel was imaged using a Dark Reader® Transilluminator and the desired bands were excised using a clean razor blade. Approximately, 4 volumes of ADB buffer were added to the gel pieces and the mixture was incubated for 5-10 minutes at 37 ºC while mixing several times during the period. PCR products were isolated from the mixture using a Zymo-25 DNA column clean up protocol. The desired PCR products were eluted from the column with 32 μL nuclease-free, purified water. 1 µL of the eluted DNA products was used for quantification with a Qubit Fluorometer and the dsDNA High Sensitivity assay.

1. **Illumina flow cell loading.**

The concentration of the desired products was determined from the Qubit Fluorometer and used to prepare an equimolar pool of 7 pM solution for cluster generation on an Illumina flow cell.

**PALM Barcoding for DNA**

**Materials:** All oligonucleotides were ordered HPLC purified from Integrated DNA Technologies Inc. (IDT). Enzymes were obtained from Invitrogen, Life Technologies, Inc., New England Biolabs, and Enzymatics Inc.

- PALM Adapters (50 µM mixture after annealing)

Adapter 1 5' **P**-GATCGGAAGAGCACACGTCT

Adapter 2 5' ACACTCTTTCCCTACACGACGCTCTTCCGATC*****T

- PALM PCR primers

PALM miRNA PCR primer 1 (25 µM) 5′AATGATACGGCGACCACCGAGATCTACACTCTTTCCCTACACGACGCTCTTCCGATC*****T

PALM miRNA PCR primer 2 (25 µM) 5′**P**-GTGACTGGAGTTCAGACGTGTGCTCTTCCGATC*****T

- PALM Barcodes (50 µM final concentration in annealed solution 50mM NaCl/10mM Tris pH 7.5)

barcode 1-P 5′ **P**-G-ATCACG-ATCTCGTATGCCGTCTTCTGCTTG/3AmMO/

barcode 1-T 5′ CAAGCAGAAGACGGCATACGAGATCGTGATC*****T

barcode 2-P 5′ **P**-G-CGATGT-ATCTCGTATGCCGTCTTCTGCTTG/3AmMO/

barcode 2-T 5′ CAAGCAGAAGACGGCATACGAGATACATCGC*****T

barcode 3-P 5′ **P**-G-TTAGGC-ATCTCGTATGCCGTCTTCTGCTTG/3AmMO/

barcode 3-T 5′ CAAGCAGAAGACGGCATACGAGATGCCTAAC*****T

barcode 4-P 5′ **P**-G-TGACCA-ATCTCGTATGCCGTCTTCTGCTTG/3AmMO/

barcode 4-T 5′ CAAGCAGAAGACGGCATACGAGATTGGTCAC*****T

barcode 5-P 5′ **P**-G-ACAGTG-ATCTCGTATGCCGTCTTCTGCTTG/3AmMO/

barcode 5-T 5′ CAAGCAGAAGACGGCATACGAGATCACTGTC*****T

barcode 6-P 5′ **P**-G-GCCAAT-ATCTCGTATGCCGTCTTCTGCTTG/3AmMO/

barcode 6-T 5′ CAAGCAGAAGACGGCATACGAGATATTGGCC*****T

barcode 7-P 5′ **P**-G-CAGATC-ATCTCGTATGCCGTCTTCTGCTTG/3AmMO/

barcode 7-T 5′ CAAGCAGAAGACGGCATACGAGATGATCTGC*****T

barcode 8-P 5′ **P**-G-ACTTGA-ATCTCGTATGCCGTCTTCTGCTTG/3AmMO/

barcode 8-T 5′ CAAGCAGAAGACGGCATACGAGATTCAAGTC*****T

barcode 9-P 5′ **P**-G-GATCAG-ATCTCGTATGCCGTCTTCTGCTTG/3AmMO/

barcode 9-T 5′ CAAGCAGAAGACGGCATACGAGATCTGATCC*****T

barcode 10-P 5′ **P**-G-TAGCTT-ATCTCGTATGCCGTCTTCTGCTTG/3AmMO/

barcode 10-T 5′ CAAGCAGAAGACGGCATACGAGATAAGCTAC*****T

barcode 11-P 5′ **P**-G-GGCTAC-ATCTCGTATGCCGTCTTCTGCTTG/3AmMO/

barcode 11-T 5′ CAAGCAGAAGACGGCATACGAGATGTAGCCC*****T

barcode 12-P 5′ **P**-G-CTTGTA-ATCTCGTATGCCGTCTTCTGCTTG/3AmMO/

barcode 12-T 5′ CAAGCAGAAGACGGCATACGAGATTACAAGC*****T

(**P =** 5′**phosphate, 3AmMO = 3**′**amine, and * = phosphorothioate linkage)**

- 10X Phosphorylation buffer, dNTP mix, T4 DNA polymerase, *E. coli* DNA Polymerase I Large (Klenow) Fragment, T4 Polynucleotide Kinase, 10X NEBuffer2, 10 μL 1 mM dATP, Klenow Fragment (3′→5′ exo-), 5X Phusion HF buffer, Phusion DNA Polymerase and Taq DNA polymerase, T4 DNA ligase, Quick Ligation Reaction buffer (New England Biolabs).
- Qubit Fluorometer, and dsDNA High Sensitivity assay, Magnetic Particle Concentrator, E-Gel EX 4% Agarose Gel, TrackIt 50 bp ladder, and 10 mM dATP, 0.5M EDTA pH8 (Invitrogen, Life Technologies, Inc.)
- Agarose Dissolving Buffer (ADB) and DNA Clean and concentrator -5 and -25 kits (Zymo Research Co.)
- T4 DNA Ligase (Rapid), using 2X Rapid Ligation Buffer (Enzymatics, Inc.)
- TE buffer pH8 (Sigma-Aldrich, Co.)
- 2100 Bioanalyzer and High Sensitivity DNA kit (Agilent Technologies)
- Agencourt AMPure XP magnetic beads (Beckman Coulter, Inc.)
- Dark Reader® transilluminator (Clare Chemical Research, Inc.)

**Methods:**

1. **End repair**

5 ng of dsDNA was mixed with 10 µl of 10X Phosphorylation buffer, 4 µl of 10 mM dNTP mix, 5 µl of T4 DNA polymerase, 1 µl of *E. coli* DNA Polymerase I, Large (Klenow) Fragment and 5µl T4 Polynucleotide Kinase in a total reaction volume of 100µl and incubated at 20°C for 30 min. The end repaired products were then isolated from the reaction using a Zymo-5 DNA column clean up protocol and eluted from the column in 32 µL nuclease-free purified water.

1. **3′ A-tailing of PCR products.**

The eluted, end repaired DNA was mixed with 5 µl 10X NEBuffer2, 10 μL 1 mM dATP and 3 µL Klenow Fragment (3′→5′ exo-). The reaction was then incubated at 37°C for 30 minutes. The A-tailed DNA was then isolated from the reaction using a Zymo-5 DNA column clean up protocol and eluted from the column in 23 µL nuclease-free purified water.

1. **Ligation of 3′- and 5′- adapters**

23 µl of eluted, A-tailed DNA was mixed with 25 µl 2X Quick Ligation Reaction Buffer, 1 µl PALM adaptors mix (15 µM each) and 1 µl Quick T4 DNA Ligase. The mixture was incubated at room temperature for 15 minutes. The DNA was then isolated from the reaction using a Zymo-5 DNA column clean up protocol and eluted from the column in 20 µL nuclease-free purified water.

1. **Gel purification**

The desired PCR products were purified by loading onto 4% Agarose EX-gel, 20 μL per lane. 2 μL of TrackIT 50bp Ladder (+ 18 µl of H_2_O) was loaded into a single lane and used as a DNA size marker. The gel was electrophoresed for approximately 20 minutes. The gel was imaged using a Dark Reader® Transilluminator and the desired product (200-250 bp) was excised using a clean razor blade. Approximately 4 volumes of Zymo ADB buffer were added to the gel pieces and the mixture was incubated for 10 minutes at 37 ºC while mixing several times during the period. PCR products were isolated from the mixture using a Zymo-5 DNA column clean up protocol. The desired PCR products were eluted from the column in 29 μL nuclease-free, purified water.

1. **PCR Amplification.**

The following reagents were combined in a PCR tube: 29 µl of size selected DNA, 10 μL 5X Phusion HF buffer, 1 uL PALM DNA PCR primer 1, 1 uL PALM DNA PCR primer 2, 1.5 µl dNTP mix, 7 μL nuclease-free purified water and 0.5 µl Phusion DNA Polymerase. The mixture was then placed in a thermocycler using the following program: 1 initial hold, 98ºC, 30 seconds; cycled 15 times, 98ºC, 10 seconds; 60ºC, 30 seconds; and 72ºC, 30 seconds; 1 final hold, 72ºC, 5 minutes. After the PCR reaction was complete, the PCR products were purified using a Zymo-25 DNA column clean up protocol and eluted from the column in 38.5 µL nuclease-free purified water.

1. **3′ A-tailing of PCR products.**

The eluted PCR products were mixed with 5 µl 10X Taq buffer, 5 μL 10 mM dATP and 1.5 µL Taq polymerase. The reaction was then incubated at 72°C for 30 minutes. The A-tailed PCR products were then isolated from the reaction using a Zymo-5 DNA column clean up protocol and eluted from the column in 21 µL nuclease-free purified water.

1. **Ligation of PALM barcodes to PCR products.**

100 ng of the eluted PCR products were mixed with 2 µL of annealed PALM barcode (50 µM) in a final volume of 23 μL and added to 25 μL of 2X Rapid Ligation buffer with 2 μL T4 DNA Ligase (Rapid) and incubated at 20°C for 30 minutes. 1 µl of 0.5 M EDTA was added to stop the ligation reaction.

1. **AMPure XP magnetic bead isolation of DNA products.**

After the ligation reaction, 90 μl of AMPure XP beads was added, mixed, and allowed to incubate at room temperature for 5 minutes. Using a Magnetic Particle Concentrator (MPC), the beads were pelleted against the wall of the tube. The tube will remained in the MCP during all subsequent wash steps. The supernatant was removed, and the beads were washed twice with 500 μL of 70% ethanol (allowed to sit for 1 minute each time during wash). The AMPure XP beads were allowed to air dry for approximately 5 minutes. Then the tube was removed from the MPC and 20 μL of TE was added and vortexed to re-suspend the beads. After a 2 minute wait step, the tube was placed back in the MPC to pellet the beads against the wall of the tube and the eluate was removed and transferred to a fresh microfuge tube.

1. **Agilent High Sensitivity DNA chip analysis and Illumina flow cell loading.**

1 µl of the eluted DNA library was run on an Agilent High Sensitivity DNA chip to determine size and concentration of the desired barcoded products. The concentration of the desired products was determined from the Agilent 2100 Bioanalyzer electropherogram and used to prepare an equimolar pool of 9 pM solution for cluster generation on an Illumina flow cell.

1. **Final (optial) gel purification**

The equimolar pool was purified by loading onto 4% Agarose EX-gel, 20 μL per lane. 5 μL of TrackIT 50bp Ladder was loaded into a single lane and used as a DNA size marker. The gel was electrophoresed for approximately 20 minutes. The gel was imaged using a Dark Reader® Transilluminator and the desired product (300-350 bp) were excised using a clean razor blade. Approximately, 4 volumes of Zymo ADB buffer were added to the gel pieces and the mixture was incubated for 5-10 minutes at 37 ºC while mixing several times during the period. PCR products were isolated from the mixture using a Zymo-5 DNA column clean up protocol. The desired PCR products were eluted from the column in 30 μL nuclease-free, purified water. This eluate was used to prepare an equimolar pool of 9 pM solution for cluster generation on an Illumina flow cell.
